# Use of the Washington Group Questions in Non-Government Programming

**DOI:** 10.3390/ijerph182111143

**Published:** 2021-10-23

**Authors:** Alex Robinson, Liem Nguyen, Fleur Smith

**Affiliations:** Nossal Institute for Global Health, University of Melbourne, Melbourne, VIC 3010, Australia; ntl1234@gmail.com (L.N.); fleur.smith@unimelb.edu.au (F.S.)

**Keywords:** disability, functioning, data, inclusion, Washington group, non-government organizations

## Abstract

The Washington Group questions (WGQ) on functioning have been widely promoted as the go-to tool for disability data collection. Designed for use by government, the WGQ have been adopted by non-government organizations (NGOs) for use in programming. However, little is known about how the WGQs are being used by NGOs or how use may be contributing to disability inclusion. Method: This paper describes exploratory research on the use of the WGQ in NGO programming. An online survey provided an overview of adoption followed by semi-structured interviews from a purposive sample to explore data collection, analysis, and use. Results: Thematic analysis showed limited inclusion outcomes directly attributable to use of the WGQ, adoption driven by individual champions rather than systematically across organizations, and challenges in data collection resulting in a wide range of prevalence rates. What information the WGQ can realistically contribute to programs was also overestimated. However, the process of using the WGQ was raising awareness on disability inclusion within program teams and communities. Conclusion: Acknowledging differences in emerging use by NGOs beyond the WGQ’s intended purpose, alongside promoting a flexible and staged approach to adoption and use in programming, may improve utility and disability inclusion outcomes over time.

## 1. Introduction

The Washington Group on Disability Statistics is a United Nations (UN) City Group established in 2001 to improve disability data collection and comparisons between countries [1]. Designed to be incorporated into government censuses or surveys, the Washington Group questions (WGQ) on functioning enable data to be disaggregated and comparisons of equality of opportunity to be made [2], for example, to identify differences in educational attainment or income levels between people with and without disability. Several question sets have been developed by the Group that are collectively known as the Washington Group questions.

The context for this study is the increasing use of the WGQ beyond government and their original intended purpose. Non-government organizations (NGOs) are using and recommending use of the questions in development and humanitarian programming [3,4,5] and for Sustainable Development Goal (SDG) and related reporting [6]. Uptake by NGOs has been accompanied by guidance on use of the questions and incorporation into standards [7,8,9]. However, we have limited knowledge on how the questions are being used in non-government programs and to what extent their use is contributing to disability inclusion. This research explores the under-researched area of how NGOs have adopted and are using the WGQ in their programs.

The WGQ use activity limitations as an indicator of functioning to identify people at risk of disability [10]. The questions ask respondents to self-report the level of difficulty they have doing everyday activities, such as walking or self-care. The questions follow a standard question and response format, use non-technical language, and aim to be based on culturally neutral activities. The design of the questions allows use in varied contexts and by enumerators with no or little knowledge of disability.

The foundational question set is the Short Set of six questions. There are also Enhanced and Extended Sets and a Child Functioning Module (CFM) designed with the UN Children’s Fund (UNICEF). A Labour Force Disability Survey Module has been developed with the International Labour Organization (ILO) and an Inclusive Education Module is in development with UNICEF. The question sets do not use the term ‘disability’ to avoid issues arising from differing understandings of disability and underreporting due to stigma [11]. The WGQ are not a diagnostic tool and do not identify impairment types or all people with disability. The purpose of the WGQ is to identify most people with disability in a population [12].

Recent studies of use in government surveys and related programs have mostly focused on issues relating to disability prevalence. A study of use in population surveys identified prevalence figures from approximately 3% to 20% [13]. Contributing factors included the choice of question set and cut-off points. The study also found the questions on self-care and communicating identified relatively few people with functioning difficulties in comparison to substituted questions on anxiety and depression. Difficulties in translating depression in the Extended Set have also been reported [14]. The ‘some difficulty’ response category stands out as capturing a large number of individuals with a lack of precision [15,16]. Research has shown that using the CFM to inform more inclusive education delivery without other data collection tools is insufficient [16,17]. A comparison of census data from a direct question on whether a person has a disability with WGQ data in North India found disability prevalence figures to be comparably low at around 2% [18]. However, how the WGQ were asked and who was asked is unclear. Despite challenges, the majority of studies find that the WGQ have the potential to contribute to improving disability inclusion.

In comparison, and despite the availability of NGO guidelines and standards, evidence on use of the WGQ in non-government programming remains largely anecdotal. Similar to the above, this exploratory study finds use of the WGQ by NGOs is resulting in varied prevalence figures. Additionally, we identify how practical issues in data collection by NGOs contribute to these variations. We note the need for more realistic consideration of what the questions may contribute to NGO programming and the need for a more flexible approach to use aligned with better identifying people with disability for inclusion in programs. While we found limited evidence of improved disability inclusion outcomes arising from use of the questions, we found processes of adoption were raising awareness and improving understandings of disability.

This research was conducted in partnership with CBM Australia under the Australian Government funded Partnership for Provision of Disability Technical Advice and Services through the Department of Foreign Affairs and Trade (DFAT) (Grant agreement number 74096). Ethics approval was from the Melbourne School of Population and Global Health Human Ethics Advisory Group (Number 1852115.1).

## 2. Materials and Methods

The study included two components. The first was an online survey to provide an overview of use of the WGQ and to identify semi-structured interview participants. All study participants were adult (18 years of age or older) professionals working in international development and/or humanitarian action. Inclusion criteria was having organizational experience of using any of the question sets in design, implementation, monitoring, or other program activity.

**Figure 1:**
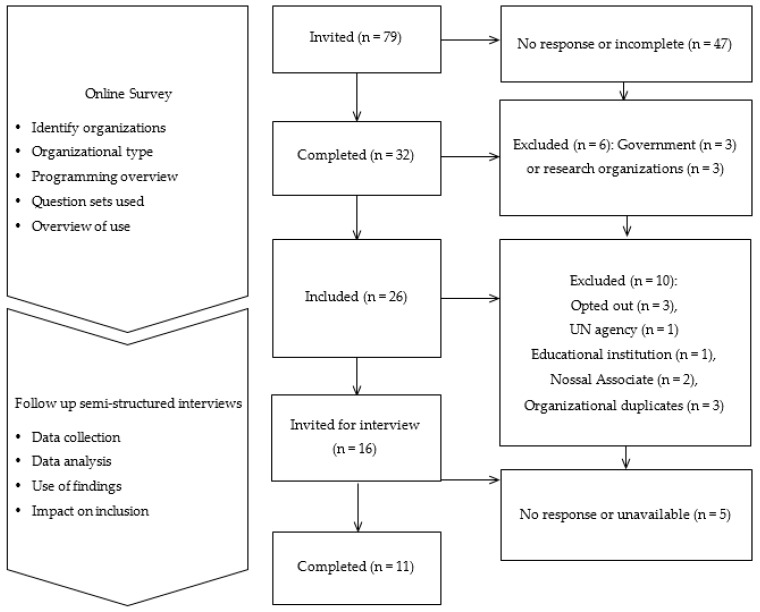
Research process.

Online survey respondents were identified via key informants from the Nossal Institute, CBM Australia, and the Australian Disability and Development Consortium (ADDC). Recruitment emails included the option to pass on the invitation to relevant partners or colleagues. Three government and three research institutions who did not use the questions in direct programming activities were excluded from the analysis of online survey data. Survey participants could opt in for participation in the follow-up interviews. Associates who had used the questions in work related to the Nossal Institute were excluded. Where there were more than one respondent from an organization, one participant was selected in consultation with the organization concerned. Participants from 11 organizations successfully completed interviews between November and December 2018. The research process, including participant numbers and key topics of enquiry, is summarized in Figure 1.

Interviews were conducted by telephone or non-video internet call for consistency and were recorded with the participant’s consent. One researcher led each interview using an interview guide and another took notes. Interviews were completed within 60 min with the exception of one slightly longer interview. Interview notes were checked against recordings and were shared with the respondent for review and approval. The final respondent-checked interview notes were imported into NVivo for coding and thematic analysis. A sample of interview notes were independently coded by two researchers. The researchers then reviewed the other’s sample notes and the code list was refined. This list was trialed by a third researcher on a further sample of interviews resulting in a final coding list. This list was used by the researchers to identify themes through an iterative process of comparison and discussion. Table 1 illustrates a sample of the final codes used and the resulting themes addressed in the results section below.

Preliminary findings were shared in a workshop with project partners and experts, including Associates excluded from the semi-structured interviews. Feedback resulted in no major changes to the identified themes; however, the importance of distinguishing uses of the questions was highlighted. Use of the questions for data disaggregation is emphasized in guidance for programs but this was not the main use identified.

As noted, this research was exploratory in nature and has limitations. We focused solely on organizations that had used the WGQ. We only interviewed one representative from each organization to avoid bias (anticipated from the online survey) from reporting on the use of a one question set across similar programs by one organization. All respondents had a prior interest or organizational focus on disability inclusion. We did not include organizations that had not used the questions and cannot speak to wider barriers to adoption. Our interview findings, which are the focus of this paper, are based on a small sample with experiences of using the questions concentrated in Southeast Asia and the Pacific. Caution should be exercised in making generalizations from the findings from this study. We are conscious of the different operational realities of humanitarian and development programming; however, we consider the findings we present to be of relevance to both.

## 3. Results

### 3.1. Online Survey

Organizational characteristics and use of the questions reported by online survey respondents are summarized in Table 2 below.

An approximately equal number of online respondents reported the questions as easy to use (*n* = 12) compared to difficult to use (*n* = 13). From these, two said the questions were very easy to use and one said very difficult. The majority of respondents (*n* = 21) felt using the questions helped with their work with most saying the questions helped a lot (*n* = 13) and the remainder saying it helped a little (*n* = 8). Only one respondent reported the questions as not being helpful with three respondents unsure. How the questions were used and were contributing to inclusion outcomes was explored in the semi-structured interviews.

### 3.2. Semi-Structured Interview Findings

Interview participants were from eight international NGOs, one national NGO, and two organizations of people with disability (OPDs). These organizations reported on work in the Pacific (*n* = 5), Southeast Asia (*n* = 5), South Asia (*n* = 1), Central Asia (*n* = 1), and Africa (*n* = 2). Findings are presented below based on the themes summarized in Section 2. Some themes have been combined under one sub-heading in the following narrative.

#### 3.2.1. Rationale for Using the Questions

No participant reported a requirement or policy mandating use of the Washington Group questions in their organization. For all international NGOs, the decision to use the questions was made at the project or country office level. It was noted that some donors had an expectation, but not a requirement, that grantees use the questions, for example DFAT and the United Kingdom’s then Department for International Development (DFID). Adoption of the questions was internally driven by motivated individuals in country offices or at headquarters.

Reasons for using the questions included consistency with the organization’s inclusion strategy or rights-based approach, growing interest in disability inclusion in the wider sector they work in, and avoiding reliance on unreliable or dated local or village government data. Almost all organizations reported only using the questions in individual projects rather than broadly across their programming. Few reported the targeted use of the questions to answer specific questions or address particular inclusion issues in projects. The simplicity of the questions was noted as positively influencing uptake and foundational Washington Group concerns, such as the need for accurate, standardized, and comparable data, were echoed by respondents.

Interview participants used the questions in a range of sectors, including education; health; nutrition; water, sanitation, and hygiene (WASH); climate change; disaster risk reduction (DRR); and humanitarian response. Project examples included improving mother and child nutrition in the Pacific, increasing access to education in Central Asia, and emergency shelter assessments during humanitarian response in Southeast Asia. The questions were used in household surveys, screening tools, participant lists, and post-disaster needs assessments.

#### 3.2.2. Guidance and Disability Inclusion in Data Collection

The majority of participants had accessed guidance from the Washington Group’s website. Published guidance for NGOs also draw on the Group’s guidelines. One organization had received training from Washington Group members and another from UNICEF on the CFM. Participants noted that individual questions, according to guidance, should not be changed, but most recognized the questions were adjusted in practice and that this was hard to monitor. Some changes were deliberate and in line with Washington Group guidance, such as changing the example for distance walked in the CFM from the length of a football pitch to between two electricity pylons. Another noted they did not mention hearing aids in the question on hearing as they were not available in their working area. Participants were aware that disability should not be mentioned when administering the questions, with one disguising their organization’s name and others introducing the survey as health, DRR, or WASH surveys. However, it was recognized that data collectors commonly mentioned disability out of habit.

NGO-focused guidance recommends the inclusion of data collectors with disability, but only one organization reported this as standard practice. For others, including people with disability in data collection was opportunistic and dependent on the availability of a known OPD. The limited availability of OPDs in working areas was considered challenging. Including data collectors with disability was reported as time intensive, and one participant noted necessary organizational systems may not be in place. For example, no budget allocation existed for reasonable accommodation, including the covering of travel costs or individual support. Organizations that had included people with disability in data collection reported positive experiences. One noted that this was critical to identifying people with disability in communities and including people with disability in trainings on use of the questions was more effective. Another noted secondary benefits with OPD participation in data collection leading to new working relationships between the OPD and local government.

The Washington Group recommends the questions be asked to all members of a household with the CFM asked to the mother or primary care giver. Not all participants had oversight of who answered the questions. It was assumed, albeit not condoned, that data collectors asked whoever was at home. Some noted asking the questions at the household level inevitably led to answers being provided by the head of household. One organization followed up with any individual with disability identified by the head of household. Another noted that, if asked, household members would only ‘bring out’ a person who had been previously identified as having a disability. One organization changed their approach to save time and now only asked the person ‘suspected’ to have a disability. Another noted their approach varied depending on available resources.

#### 3.2.3. Integration in Data Collection Tools and Modifications

Most participants used the WGQ alongside other questions. Only one organization, working in WASH, reported using the questions in a large household survey with multiple other questions. One organization used the Short Set as a stand-alone tool with no additional questions. In general, few questions were asked alongside the WGQ. Questions on gender were usually included with some respondents including questions on use of assistive products, transportation needs, or allergies and other health issues. One organization asked whether the person identified as being a person with a disability after the WGQ had been asked.

Some modifications to the Short Set were reported with two organizations adding a question on hand function. A WASH actor added a question on turning on taps. Another added a hand function question for use in livelihoods interventions that was adopted from a livelihood survey form used by the national statistics office. One of these organizations recognized that the question on self-care addresses hand function; however, they considered this insufficient as people adapted to difficulties in dressing and washing and underreported difficulties. No organizations reported using less than the full six questions in the Short Set.

#### 3.2.4. Translations

The availability of translations was reported as an issue. Participants noted guidance on translation would be helpful and were not aware of the translation protocol available from the Washington Group [19]. Few organizations translated the questions themselves, with most using translations from other NGOs or government. Often, only translations of the Short Set were available and it was noted that government translations may deviate from the original English language version. Back translation to check accuracy was considered desirable but resource intensive, particularly in humanitarian settings. No other method of validating translations was reported. The need for translations into local languages and not just the national language was emphasized.

Translation challenges included differentiating between ‘remembering’ and ‘concentrating’ in question six and between the ‘some difficulty’ and ‘a lot of difficulty’ response categories. Question five on self-care was considered hard to translate by several participants. The issue of ‘cultural overlay’ and what is ‘normal behavior’ for children was noted as an issue in the CFM. As a result of these issues, data collectors often explain and translate on-the-fly. This could be to add context, be due to working from written English survey forms, or the need to translate from a national language into a local language or dialect. Training data collectors to anticipate difficulties and to provide standardized explanations, such as drawing on optional questions in the Extended Set, was not usual practice.

#### 3.2.5. Data Analysis and Cut off Points

Analysis of data was descriptive and based on simple counts of the functioning difficulty recorded. Some participants disaggregated the disability data collected by gender and age. While simple counts and determining percentages across domains did not present issues, respondents felt they lacked knowledge on, or clear guidance for, more detailed comparative analysis. Participants found guidance on analysis from the Washington Group website to be unclear [20]. Overall, participants did not feel confident in analysis and although more detailed analysis was considered desirable it was not clear to what end such analysis would be applied. Most organizations conducted analysis in the country office with a small number of international NGOs sending data to head office or a central monitoring and evaluation specialist.

Participants were aware the ‘a lot of difficulty’ cut off point is recommended for use in censuses and national surveys to identify people with disability. Several participants noted including ‘some difficulty’ responses was important for increasing program participation. Another reflected on the subjective nature of responses and reported some senior OPD members with disability would answer ‘some difficulty’ when asked. One participant said including ‘some difficulty’ responses had been an ‘eye opener’, as it identified people they had not considered before. One organization counted people who answered some difficulty in at least two domains as a person with disability. Several organizations considered it a requirement to use the ‘a lot of difficulty’ cut off point; however, one reflected that this probably meant they were missing people with disabilities.

#### 3.2.6. Identification, Participation, and Screening

The main reported use of data was to identify people with disability for inclusion in program activities; however, this was not without issues. One participant noted the data confirmed what they already knew; that is, that there were people with disability in their working area. While one noted consistently higher figures than from local government data, another questioned the high prevalence figures they were finding. Another noted they were finding 1% to 2% prevalence when using the CFM and were concerned this did not reflect the reality on the ground. Inadequate training of enumerators was noted as a contributing factor. One participant noted prevalence data from work in different countries ranged from 1%, attributed to data collectors persisting in first asking who in the household had a disability, to what they considered a more realistic 15%.

The questions were reported as being used to measure the participation of people with disability in program activities. This was often limited to measuring the attendance of people with disability in an activity, such as a training or workshop. No examples of assessing the extent of participation were provided, including what proportion of people with disability from the overall community took part in activities. Several respondents used the questions to inform accessibility measures for workshops and program activities. Examples included conducting preparatory activities with participants with disability, ensuring physical accessibility of venues, and preparing information in alternative formats. One respondent reflected that the need for improved accessibility exists, and is independent from, whether this data is collected or not.

Concrete examples of how Washington Group data was contributing to improved disability inclusion outcomes were limited. One participant observed that despite using the questions, evidence of increased participation of people with disability in their programs remained anecdotal. One noted it was too early to tell if using the questions was increasing participation of people with a disability or not. Two organizations used the questions as a screening tool for more in-depth follow up. One used the Short Set to rapidly screen households for people with disability in new working areas. The other used the CFM to screen children with disability in schools prior to individual assessments. This was reported as aiding the development of teacher training materials and improving classroom practice. Washington Group data was also used for advocacy, including to deliver ‘stronger’ messaging to government. As discussed below, the most obvious impact was how the process of using the questions was contributing to change.

## 4. Discussion

### 4.1. Use of the Questions in NGO Programming

The online survey showed the WGQ are being used in a range of programming contexts; however, the interviews suggested applications of the questions were narrow and their perceived utility less clear. As noted, the WGQ have been promoted as a tool to disaggregate data by disability. This has been reiterated in guidance for NGO programming. Of course, for this to be possible the questions allow people with disability, or at risk of disability, to be identified. Interview findings show use of the questions was directed towards identification as an end goal, rather than as a step towards disaggregation.

NGOs, in the main, are not in the business of compiling large data sets for disaggregation and more complex analysis. An exception may be more sophisticated monitoring and evaluation of large programs. While online survey responses indicated use in monitoring and evaluation, this was not reflected in interviews. From interviews, the primary purpose of using the questions was the identification of people with a disability to increase their participation in program activities. This was, in the main, via rapid data collection using easy to administer tools. Efforts were also localized and limited in scope compared to the studies of prevalence previously noted. Acknowledging the distinction between identification and disaggregation would assist in tailoring guidance to improve use by NGOs.

Examples of use that showed clearer program benefits were as a screening tool with subsequent follow up. If the aim is to increase participation in program activities by identifying people who may otherwise be excluded, it is appropriate to use the ‘some difficulty’ rather than ‘a lot of difficulty’ cut off. While the Washington Group recommends the latter for disaggregation of data from censuses and national surveys, the Group recognizes disability is experienced on a continuum [12]. As noted, the ‘some difficulty’ response category captures a wide range of difficulty levels, and including the ‘some difficulty’ response can minimize the risk of missing potential program participants.

In terms of analysis, sophisticated methods are not necessarily needed for identification purposes and simple counts will suffice. Interview participants questioned whether their analytical skills were sufficient and suspected they should be doing more. However, this depends on the purpose of analysis and the desired programming goals. Participants faced no difficulties with the analysis required to identify the number of people with disability in their programming area. In summary, consideration of, and clarity on, end use should be the driver of guidance and resource allocation to support use of the questions in programming.

### 4.2. The Need for Realistic Expectations

As noted, the WGQ are not diagnostic, and identification of people with disability does not equate to identifying needs. The questions also do not in themselves provide information on the barriers individuals with disability face or on the access requirements an individual may have. Clarity in guidance on how the questions may or may not contribute to disability inclusion in programs would be helpful. From interviews, it was evident that expectations of what the questions might contribute to programming were often higher than what may be realistically achieved. There was also confusion over the purpose of the individual questions in the Short Set in particular. Individual questions allow disaggregation by a limited number of activity limitations, such as difficulty seeing or walking, and the response options provide some indication of the level of difficulty experienced. However, this is not their primary purpose.

The purpose of the individual questions is to contribute to answering the overarching yes-no question of who has a disability. They are a means to an end that allow disaggregation of data sets and inequality of opportunities to be identified. Similarly, use of the questions in programming needs to be considered as one part of a disability data collection process. The Washington Group questions cannot answer all disability data questions. For example, ensuring reasonable accommodation will still require consultation with the individual concerned. Asking the Short Set to interpret accessibility needs prior to, for example, a workshop is cumbersome in comparison to asking a direct question on accessibility needs. The benefits of asking OPD members the Washington Group questions are not clear when a direct question would suffice. A more discerning approach to what the questions can and cannot contribute to programming, and when, as part of a broader disability data collection process may be more effective.

### 4.3. Flexibility in Use

Early iterations of the Short Set and UN census guidance note four core or essential questions and two additional or supplementary questions; that is, questions five on self-care and six on communicating are preferred [21,22]. The Washington Group notes that if the question on self-care is culturally inappropriate, it can be omitted [23]. From an analysis of 2013 United States National Health Interview Survey data, the Group found a prevalence rate of 9.5% using all six questions and 9.3% using the first four [23]. Comparisons of using four or six questions in Vietnam found similarly low differences [24] and recent research, as noted, confirmed this [13]. We understand the argument that ensuring all six questions are asked may reduce the risk that anyone is missed. One participant strongly felt all six questions in the Short Set should be used or none at all. However, questions five and six presented particular issues in terms of translation and use. That all six questions are required assumes the questions are asked correctly and data collection processes are effective. The range of prevalence rates reported by participants and in the studies outlined earlier suggests this is not yet the case.

In NGO programming, there is room to consider the staged adoption of the questions. The effective use of the first four questions of the Short Set may identify more people at risk of disability than six questions used poorly. As teams and data collectors become more confident using the questions, the remaining two could be added. The time allocated to training data collectors was also reported as limited in the main. This ranged from half a day in a humanitarian context to an exception of five days, with three days being the norm. Persisting issues, including directly mentioning disability and on-the-fly explanations, suggests that this is insufficient. Particularly when we consider the time required to understand the rationale behind the questions and why disability should not be mentioned; strategies for dealing with known issues and explanations if they are to be provided; and ensuring sufficient time for repeat practice and reflection. Staged adoption of the questions alongside increased attention to avoiding known data collection issues, such as reducing the need for on-the-fly translations, may be more beneficial over time.

### 4.4. Processes Rather Than Outcomes

Despite the challenges organizations are facing using the questions, there are notable positives. Use of the questions to better identify people with disability for inclusion in programs is undoubtedly important. Currently, the WGQ are arguably the most effective and resource efficient tool we have for this purpose. While we did not identify concrete examples of positive impact for people with disability resulting from use in programming, the process of using the questions was contributing to positive change. It was how the WGQ were influencing processes rather than direct outcomes that stood out. That use of the WGQ was changing attitudes was also noted by a Leonard Cheshire and Humanity and Inclusion study conducted at a similar time to this research [25]. While we did not find evidence of widespread institutional uptake or cultural change across organizations, we agree this suggests promise.

The process of adoption and use of the questions was reported as generating new discussions and raising awareness. Use of the questions was elevating the profile of disability inclusion as an issue of focus within offices and projects. Through incorporating the WGQ in their work, teams were learning more about disability and disability inclusion. Similar benefits were also noted in, and through engagement with, communities where organizations worked. Using the questions in communities was generating conversations about disability that program teams had not had before. The process of using the questions, however imperfectly that may be, was reported as contributing to changing attitudes and addressing stigma. In terms of disability inclusive programming, and while acknowledging the challenges of attributing impact, any such outcomes can only be desirable.

## 5. Conclusions

Our research drew on a small sample of respondents and we are cautious of making broad assumptions. We deliberately focused on organizations that had used the questions and the majority of these had a prior interest in disability inclusion. It is noteworthy that early and motivated adopters faced difficulties using the questions and in assessing what the questions could contribute. If these organizations are facing challenges, it is reasonable to expect organizations new to disability inclusion to face further barriers to adopting and sustaining use of the questions in their work.

In terms of disability inclusion, uptake of the WGQ in non-government programming is encouraging. Efforts to identify and increase the participation of people at risk of disability in development and humanitarian programming are clearly welcome and much needed. While we were not able to identify direct outcomes for people with disability arising from use of the questions, the process of using the questions was contributing to change. Examples of positive change were not only within offices and program teams but, for some, extended to host communities.

In NGO guidance, it is important to acknowledge that end uses will likely differ from the Washington Group’s original purpose. As noted, flexibility is required. If the aim is advocacy to government, it may be preferable to use the questions in line with the Group’s recommended protocols to ensure consistency. For other programming purposes and aims it may not. Differences should be recognized in guidance and considered in program design. For example, inclusion of ‘some difficulty’ in analysis may increase utility. A staged introduction of use of the Short Set may allow more time for improving rigor in data collection, particularly with teams unfamiliar with disability. While using the full six questions may be preferred, ineffective use of any of the questions is unhelpful.

A flexible but considered approach to how the WGQ are used in NGO programming may help better demonstrate possible contributions to impact, an area that warrants further inquiry. In turn, this may promote adoption beyond individual champions to across institutions and by organizations that are yet to actively consider disability inclusion. Currently, it is this process of adopting the questions that shows the most promise for improving disability inclusion in NGO programming.

## Figures and Tables

**Table 1:** Overview of interview topics, codes, and themes.

Topics	Codes (Not Exhaustive)	Themes
Data collection Data analysis Use of findings Impact on inclusion Challenges/opportunities	Adaptation Acceptability Alternative data Consistency in use Comparability of findings Disaggregation WG protocol Direct question on disability Enumerators Funding and costs Identification Institutionalization Organizations of People with Disability and other stakeholders Impact Mainstreaming Motivation Participation Quality control Research tools Response categories Sampling Screening Sharing data Stigma and prejudice Strategic use Training guidance and support	Rationale for use Disability inclusion in data collection Guidance and adaptation of WGQ for program use Integration of WGQ in data collection tools Modification of question sets Translations and availability Choice of cut off points for analysis Application of WGQ: identification Application of WGQ: participation Application of WGQ: screening

**Table 2:** Organizational characteristics and use of the WGQ.

Characteristics and Use of the WGQ		Frequency
Type of organization	International NGOs (INGOs)	11
	National NGOs	5
	Organizations of People with Disability	4
	Others (e.g., managing contractor, consultancy firms)	6
Disability focused organization	Yes	20
	No (4 INGOs & 2 other types of organization)	6
Size of organization	<10 people	6
	10 to 29 people	5
	30 to 99 people	5
	>100 people	7
	Missing data	3
Geographical areas the questions were used ^a^	Pacific	5
	South Asia	5
	Southeast Asia	4
	Other parts of Asia	2
	Middle East & North Africa	2
	Other parts of Africa	5
	America & Europe	1
Disability question sets used ^a^	WG Short Set	22
	WG Extended Set	7
	WG/UNICEF Child Functioning Module	5
	Rapid Assessment of Disability Toolkit (RAD)	4
Purpose of using the questions ^a^	Program design	9
	Implementation	18
	Monitoring & Evaluation	19
	Advocacy	9
Programming areas ^a^	Education & Training	12
	Disaster Risk Reduction (DRR)	9
	Humanitarian Response	6
	Work & Livelihoods	6
	Human Rights (advocacy)	6
	Health	5
	Water Sanitation & Hygiene (WASH)	5
	Others (e.g., elimination of violence against women, child protection)	7

Note: ^a^ = Multiple choices.

## Data Availability

Data from this research is not available for ethical reasons as permission for extended use and/or sharing of data was not sought from participants.
